# Formula for Combined Administration of Cod-Liver Oil and Phosphorus

**Published:** 1876-01

**Authors:** 


Formula for the Combined Administration of Cod-liver Oil and Phosphorus.—Dr. Edward C. Mourn (*New York Med. Record,* September 18th) has employed the following mixture with the happiest results, patients taking it readily who could not bear the plain cod-liver oil at all: ℞.—Yolks of three eggs; cod-liver oil, 8 ounces; sherry wine, 4 ounces; phosphoric acid, simple syrup, of each an ounce; bitter almond water, 8 ounces; rectified spirit, a drachm. Rub the eggs up in a mortar, adding the oil spoonful by spoonful. Last of all, add the phosphoric acid.—*American Med. Weekly.*

